# Stakeholder perspectives on the impact of COVID-19 on oncology services: a qualitative study

**DOI:** 10.1007/s00520-023-07916-y

**Published:** 2023-07-24

**Authors:** Phyllis Butow, Polly E. Havard, Zoe Butt, Ilona Juraskova, Louise Sharpe, Haryana Dhillon, Lisa Beatty, Philip Beale, Maria Cigolini, Brian Kelly, Raymond J. Chan, Laura Kirsten, Megan C. Best, Joanne Shaw

**Affiliations:** 1 Psycho-Oncology Co-operative Research Group (PoCoG), School of Psychology, University of Sydney, Camperdown, NSW 2006 Australia; 2 Flinders University, Órama Institute, College of Education, Psychology & Social Work, Adelaide, SA 5001 Australia; 3 Concord Clinical School, Faculty of Medicine and Health, University of Sydney, Camperdown, NSW 2006 Australia; 4 Department of Palliative Medicine, Royal Prince Alfred Hospital, Missenden Road, Camperdown, NSW 2050 Australia; 5 School of Medicine and Public Health, University of Newcastle, Callaghan, NSW 2308 Australia; 6 Caring Futures Institute, College of Nursing and Health Sciences, Flinders University, Bedford Park, SA 5042 Australia; 7 Nepean Cancer Care Centre, Penrith, NSW 2751 Australia; 8 Institute for Ethics and Society, University of Notre Dame, Broadway, Sydney, NSW 2007 Australia

**Keywords:** COVID-19, Cancer, Oncology, Health service change, Qualitative

## Abstract

**Background:**

As COVID-19 spread across the globe, cancer services were required to rapidly pivot to minimise risks without compromising outcomes for patients or staff. The aim of this study was to document changes to oncology services as a result of COVID-19 from the perspectives of both providers and receivers of care during the initial phase of the pandemic.

**Methods:**

Participants were recruited between June and December 2020 through an email invitation via professional or consumer organisations, two hospital-based oncology services and snowballing. Semi-structured interviews focused on health service changes and their impacts, which were then analysed by thematic analysis.

**Results:**

Thirty-two patients, 16 carers and 29 health professionals were recruited. Fifteen patients (*n* = 47%) had localised disease, and 19 (*n* = 59%) were currently receiving treatment. Oncology staff included oncologists, palliative care physicians, nurses, allied health and psychosocial practitioners. Four themes arose from the data: safety, increased stress and burnout, communication challenges and quality of cancer care.

**Conclusions:**

There is an ongoing need for cancer-specific information from a single, trusted source to inform medical practitioners and patients/carers. More data are required to inform evidence-based guidelines for cancer care during future pandemics. All stakeholders require ongoing support to avoid stress and burnout.

**Supplementary Information:**

The online version contains supplementary material available at 10.1007/s00520-023-07916-y.

## Background

In 2020, life changed worldwide as the COVID-19 pandemic spread rapidly [1]. While everyone is at risk of COVID-19, the risk of dying from the disease is significantly higher for those who are older or have compromised immune function or chronic disease, especially those involving the respiratory system. Cancer patients are one such vulnerable group [2]. Health professionals (HPs) exposed to people who are COVID-19-positive are also at greater risk of developing COVID-19.

The need to protect patients and HPs from contracting COVID-19 was rapidly recognised within cancer services. Fast-track changes were made to increase the use of telehealth to minimise risks associated with hospital attendance [3]. Treatment delays were considered to avoid cancer treatment-induced immuno-suppression [4]. Hospitals imposed rules excluding or severely limiting family members from accompanying or visiting patients. Throughout the pandemic, some screening services temporarily ceased (e.g. breast screening and colonoscopy services). Meanwhile, the pressure to develop significant capacity (space, resources and staff) to manage COVID-19 cases began competing for hospital resources. In Australia, some oncology services were shifted to private hospitals to free space in public hospitals for COVID-19 wards.

Studies are reporting significant impacts of COVID-19 on the experiences of cancer patients, carers and HPs. Elevated moral distress, stress and burnout have been documented in the oncology workforce [5, 6] and heightened anxiety and depressive symptoms in patients and carers [7, 8]. However, some changes were welcomed by HPs and patients [3], suggesting that close examination of the benefits and costs of COVID-19-related changes is worthwhile, to determine what might be usefully retained post-pandemic.

Most studies of stakeholder experiences to date have been conducted in the USA, Europe and Asia where COVID-19 outbreaks were very severe, overwhelming the health system. Australia initially experienced smaller COVID-19 outbreaks, with a lesser impact on cancer services [4]. Thus, Australia provides an interesting case study of how oncology services with more time to respond to COVID-19 have pivoted and the impact of those changes.

The aim of this study was to document experienced changes to oncology services as a result of COVID-19 by both providers and receivers of that care and the impacts of those changes in the acute phase when COVID-19 emerged.

## Methods

This was a qualitative study involving semi-structured interview data, collected from June to December 2020. We have previously reported the emotional impact of COVID-19 on study participants [9]. This paper focuses on cancer service change and how that was received by providers and receivers.

## Participants

Eligible participants were as follows: (a) adult cancer patients (18 years or over) currently receiving or within 6 months of treatment; (b) family carers of an eligible cancer patient; and (c) oncology HPs (including surgeons, oncology and palliative care doctors and nurses, allied health workers, chaplains and psycho-oncology staff). For patients and carers, non-English speaking or incapacity to give informed consent were exclusion criteria.

## Recruitment and study procedure

Participants were recruited through an email invitation via professional or consumer organisations, two hospital-based oncology services and snowballing (whereby health professional participants recommended the study to colleagues by forwarding the introductory email) [10]. A participant information sheet, consent form and brief survey were accessible via a link embedded in the email. The research team contacted interested participants to schedule a telephone interview. Diversity in age, gender and residence (urban or regional/rural) was sought where possible. Recruitment continued until theoretical saturation (i.e. no new emerging themes) was reached within each stakeholder group.

## Data collection

Demographic, clinical and employment characteristics were elicited at baseline. Semi-structured telephone interviews (30–45 min in length) were conducted, exploring experiences and impact of COVID-19-related changes to cancer services. Interview questions are shown in Table 1.

**Table 1:** Interview questions

Healthcare professionals (all)	
What has changed for you as an oncology healthcare professional, since the COVID-19 pandemic began? Prompt: concerns, role, clinical care (telehealth), health system changes, patient and family concerns and behaviour	
What worries you the most about COVID-19, as an Oncology healthcare professional?	
What COVID-19 policies have been put into place by your Oncology service? What about in your health service as a whole?	
How are you communicating with your patients about COVID-19?	
Has the COVID-19 pandemic influenced decisions you and your patients are making regarding clinical care and follow-up? Can you give me a few examples? What issues were raised? How were the risks weighed up? What decisions were made in the end? Were you uncomfortable with any of these decisions? If so, were you able to resolve that?	
What areas of life have most changed for you since COVID-19? What are you most afraid of in relation to COVID-19?	
How are you and your colleagues coping personally with COVID-19 concerns? What has helped, made it more difficult?	
How are your patients and their families responding to COVID-19 coping? What support would benefit them?	
Would you like to say anything else re being a health professional during COVID-19?	
Patients*	
As a cancer patient/survivor, can you tell me what has changed for you since the COVID-19 pandemic began? Prompt: Concerns, clinical care, family support, your behaviour	
Where have you been getting most of your information about COVID-19?	
What communication have you had (if any) from your oncology team, about COVID-19?	
What worries you the most about COVID-19, as a cancer patient/survivor?	
Have you made any decisions regarding your cancer care or follow-up since COVID-19? If yes, did you consider COVID-19 while you were deciding what to do? Prompt: What COVID-19 issues did you consider? What did your Oncology team advise? What did you end up deciding?	
How are you coping with COVID-19 concerns? What has helped or hindered?	
What support or information regarding COVID-19 would you like from your treatment team? Or for others?	
Is there anything else you would like to say about being a cancer patient/survivor during the COVID-19 pandemic?	

*Family members’ interview identical with small adaptations

## Planned analyses

Demographic descriptive statistics were generated for all participant groups. Interviews were audio-recorded, transcribed verbatim, anonymised, uploaded to NVIVO 12 and subjected to thematic analysis using a framework analysis approach [11]. Line-by-line coding was conducted on three transcripts by the research team to develop the preliminary coding framework, which was iteratively refined following a review of subsequent transcripts. Over-arching themes and sub-themes were developed to summarise the data. Differences in researcher interpretation of the data were resolved through discussion. Comparison of themes arising from different participant groups and in those with higher versus lower anxiety/depression/ stress on quantitative measures were made. Consolidated criteria for reporting qualitative research (COREQ) were used to guide reporting [12].

## Results

Thirty-two patients (mean age of 61, 23 females), participated (see Table 2). The majority had breast (*n* = 12) or prostate (*n* = 9) cancer. Fifteen had localised disease, and 19 were currently receiving treatment. Sixteen carers (mean age of 57, 15 females) participated, most of whom (*n* = 12) were a spouse/partner.

**Table 2:** Cohort characteristics

	Patients (*N* = 32)	Carers (*N* = 16)	Health professionals (*N* = 29)
Variable	*N* (%) *	*N* (%)	*N* (%)
Gender			
Male	9 (28)	1 (6)	2 (7)
Female	23 (72)	15 (94)	27 (93)
Age in years	Mean = 61	Mean = 57	Mean = 48
<40	4 (13)	2 (13)	9 (31)
41–50	2 (6)	2 (13)	6 (21)
51–60	6 (19)	4 (25)	8 (27)
61–70	14 (44)	7 (44)	4 (14)
71–80	6 (19)	1 (6)	1 (3)
Born in Australia	21 (66)	12 (75)	25 (86)
English spoken at home	32 (100)	14 (88)	27 (93)
Level of education			
School	5 (16)	1 (6)	0
Technical certificate	8 (25)	2 (13)	1 (3)
Undergraduate degree	9 (28)	4 (25)	4 (14)
Postgraduate degree	10 (31)	9 (56)	24 (83)
Employment			
Employed	9 (29)	7 (44)	28 (96)
Retired	19 (59)	6 (38)	0
Unable to work	2 (6)	6	0
Unemployed	2 (6)	2 (13)	0
Student	0	1 (6)	1 (3)
Time since diagnosis			
<1 year	12 (38)		
1–5 years	17 (53)		
>5 years	3 (9)		
Cancer type			
Breast	12 (38)		
Prostate	9 (28)		
Lung	3 (9)		
Others	8 (25)		
Cancer stage			
Local	15 (47)		
Locally advanced	5 (16)		
Metastatic	9 (28)		
Others	3 (9)		
Currently on treatment	19 (59)		
Relationship with patient			
Child		2 (13)	
Spouse/partner		12 (75)	
Parent		1 (6)	
Lives with patient		13 (81)	
Has own medical condition		7 (44)	
Profession			
Oncologist/palliative care			8 (28)
Nurse			10 (34)
Psycho-oncology			9 (30)
Allied health			2 (7)
Public or private practice			
Public			18 (62)
Private			3 (10)
Mixed			7 (24)

*Numbers vary due to a small amount of missing data

Twenty-nine HPs (mean age of 48, 27 females) participated. They included 10 nurses, 6 medical oncologists, 9 psycho-oncology staff, 1 palliative care physician and 2 allied health practitioners (a physiotherapist and genetic counsellor).

Qualitative analysis yielded four themes related to the impact of COVID-19-related oncology service changes: safety, increased stress and burnout, communication challenges and quality of cancer care. Below these themes are explored, with characteristic quotes identified by source (patient (P), carer (C), or health professional (HP)) and participant ID. Additional quotes are presented in Supplementary Table 1.

### Safety

Patients and carers observed many altered procedures in hospitals to reduce the risk of COVID-19 infection, including temperature checks, screening questions, masks, personal protection equipment (PPE), extra cleaning and hand sanitizer. Participants felt comforted and reassured by these precautions.

... The doctor said you’re in a safe area. Try and hold onto that. (P321)

Only one patient felt hospital procedures were not sufficiently rigorous to ensure safety:

We didn’t have temperatures taken or anything… I thought a little bit casual. (P340)

Despite infection control procedures, HPs worried that patients/carers were prioritising COVID-19 safety over seeking diagnostic and treatment services, with downstream consequences of poorer outcomes.

It worries me there’s all these patients [with] cancers getting upstaged while they sit at home… are we going to see a flood of advanced malignancy in six months’ time?… there’s been a couple of patients who have come in with the worst cancer I’ve ever seen. (HP148)

### Increased stress and burnout

The exclusion of carers from hospitals left many patients feeling stressed and isolated. Social distancing reduced opportunities to share experiences with other cancer patients; telehealth reduced human contact. Carers were also impacted, as noted by patients and HPs.

Their [carers’] anxiety levels are often much higher than the person who has cancer… because they don’t have the care team …, interacting with them all the time, they’re somewhat left out of it. (HP127)

Many HPs commented they were grappling with bigger workloads and longer working hours, as they processed ever-changing COVID-19-related information, established new guidelines and procedures to minimise risk and communicated these to patients and carers. Different consent and scheduling procedures for remote consultations added layers of administration. Several HPs mentioned online work required intense listening to pick up cues, was more fatiguing and provided less job satisfaction.

I found I couldn’t do any more than three back-to-back [telehealth] sessions because I was just exhausted. (HP105)

… we’ve seen a lot of that shifting to email. But… it takes a lot longer to answer an email than it does to have a conversation with someone… And so most clinicians [are] staying back till 6.30 at night, which means their own families are suffering and their own work-life balance and wellbeing starts to suffer. (HP102)

HPs discussed the strains of being short-staffed when staff stayed away from work due to symptoms or while awaiting COVID-19 test results or when redeployed to other tasks.

So suddenly there’s five team members out of our ward…and we are under stress. (HP146)

### Communication challenges

#### Information

Many patients felt they had received general rather than personalised information from HPs about their COVID-19 risk, such as text messages and signage about hospital changes, which were helpful but did not assuage their personal fears. They wanted individualised information regarding the interaction between their cancer and COVID-19. In contrast, some patients appreciated care and advice received from HPs.

What would have been helpful is probably the stuff that they don’t know... a known level of elevated risk or not. (C402)

Many HPs noted the rapidly evolving and conflicting messaging for HPs. Several desired more guidance about issues such as medico-legal and ethical considerations associated with treatment decision-making and telehealth, including scheduling, what was appropriate for new versus established patients, and how billing worked in this context. Others sought information about what and how to communicate to patients regarding how cancer and COVID-19 interact, as well as how to explain hospital policies and rules to limit infection.

…at the time we were all fumbling… there was a lot of information gaps, but (no) information to fill these gaps (HP104)

And the ethical and medico-legal aspects of it… Should I dose reduce chemotherapy because they’re at greater risk?” (HP106)

All participants wanted simple, clear, consistent messaging that was not confusing, preferably from a single, reputable, government source such as Cancer Australia or the State government’s Department of Health.

#### Communication quality

Most patients praised their HPs for behaving professionally despite the stresses of COVID-19 and reported they had good care and responsive HPs. However, some patients and carers discussed experiencing a lack of holistic care and felt doctors were too busy to share information.

People just want to get her off the phone call or the consultation. And so…she doesn’t feel like she can ask questions. (C408)

Similarly, some HPs felt communication had suffered during the pandemic. Mask-wearing and lack of physical touch were considered “dehumani*zin*g” (HP149).

And then… they stick masks on us… In delivering really difficult news, suddenly you just get to see my eyes... And…seeing distress on their face, I can’t give them a hug. (HP149)

One HP observed bad news being delivered by someone the patient did not know because of scheduling changes. HPs and carers noted nurses could not attend appointments to provide follow-up information when patients had questions, because of social distancing rules. However, others felt staff were putting in extra effort to support patients in this difficult time.

Many patients, carers and HPs noted telehealth was more impersonal and made it harder to observe non-verbal cues (even with video), ask questions and build rapport, observe physical changes and share and discuss results, requiring good communication skills in all parties and reliable internet. Several HPs commented that telehealth took away “the art of medicine” (HP102). One patient felt information conveyed in telehealth appointments was harder to remember.

I’m trying to have a serious conversation about my treatment plan… and the internet is cutting out and I can’t see the medical professional I’m talking to… that makes what is already a fairly fraught experience even more so… I never felt like I had fully understood everything. (P328)

Despite this, most patients and HPs agreed telehealth was sufficient given the circumstances and convenient in terms of reduced travel.

…I’ve actually quite liked it [telehealth] because we’re able to offer a bit more support to regional patients, patients who can’t get into the hospital for other reasons. (HP105)

Several HPs observed that video was better than phone telehealth, although others found video too difficult to set up and manage for many patients. One HP observed patients were more inclined to *open up* over the phone because they had *greater anonymity* through that medium *(HP104)*. HPs overcame barriers by dialling in interpreters and asking patients to send them photos of symptoms to supplement the phone call.

### Quality of care

Most patients perceived they had not experienced cancer treatment changes due to COVID-19, but some were concerned about future changes, such as elective surgery (e.g. breast reconstruction) being stopped. Some patients were critical of delays to screening, testing and diagnosis as well as some treatments due to COVID-19. Some were concerned about treatment delays while awaiting COVID-19 test results (this was also noted by HPs) and felt communication about such delays was poor. Another patient described delays in contact from care coordinators. However, some carers noted they had been reassured by the oncologist saying any delays were largely immaterial and would not impact outcomes.

He used to have immunotherapy before the operation every two weeks. And after the operation, the oncologist… said a lot of people are changing it to monthly because of Covid-19... But he said, either way, there’s no real harm. (C407)

Most HPs did report some changes to treatment schedules, and some expressed concern about negative impacts.

We’ve adjusted some of the treatments we’ve given, or the follow up… we’ve backed off on some of the more intensive regimens... in some cases you might be worried whether patients are going to have a more detrimental outcome. (HP148)

Most changes aimed to reduce infection vulnerability. For example, thresholds for delaying or avoiding chemotherapy were reconsidered based on factors such as patient age and when benefits of treatment were less clear. Several HPs noted changes to treatment type such as offering oral versus systemic chemotherapy which patients could have at home, or changes to the patient’s treatment schedule to reduce hospital attendances. Some noted minor investigations were stopped completely, particularly lung function tests due to *concern about spread from saliva and breath (HP100).* However, HPs largely felt nothing drastic was changed and that patients were not being denied treatment or given a lower priority due to COVID-19, despite this being a concern for HPs and something they prepared for. Some HPs noted that rates of unnecessary treatment had probably reduced, as it was easier to reach such decisions on the basis of COVID-19.

And I think before COVID–sometimes we do give chemotherapy that maybe we shouldn’t, and I’ve been doing much less of that. And that’s probably a good thing (HP148)

HPs took a shared decision-making approach with patients while trying to realistically present the pros and cons of alternative approaches.

… we always try and present the options to them….but given the complexities associated with COVID-19, we [are more likely to say we] think the risks of giving you the combination are too high, or that the risks outweigh the benefits. (HP126)

Regarding the general quality of care, some patients appreciated reduced wait times for appointments which accompanied changes implemented to reduce the number of people in clinic.

It actually went a lot smoother than previous ones because there didn’t seem to be any wait times in the consulting rooms. (P304)

Negative perceptions of hospital-level changes most commonly related to not being able to have a support person attending appointments, which meant patients lost the benefit of having someone else there to ask questions, take in information and receive instruction in home care. One patient discussed her disappointment that all the peripheral integrative services in hospital had been shut down, reducing the quality of her hospital experience. Another described an upsetting move to a different hospital when her original hospital became a COVID-19-designated centre.

Some HPs were also concerned that the quality of general care had reduced due to stress, time pressures, stretched resources and lack of face-to-face contact.

A lot of other medical staff, and nursing… I think they’ve lost a lot of their compassion towards our patients. (HP142)

Patients and HPs were also concerned that cancer patients would be a low priority for COVID-19 treatment should they contract it. Some HPs were also worried about being unable to provide optimal care.

…one patient arrested, and they couldn’t do anything for them while they were dying … because CPR was not allowed in that ward and neither was going close and giving oxygen [until PPE was worn]… (HP508)

That was difficult, because… the nurses are all at work, but the doctors weren’t. We did have quite a few patients fall through the cracks and a few toxicities and a few issues around that. (HP142)

## Discussion

This study documented the experiences of patients, carers and HPs regarding changes to cancer care as a result of COVID-19. While there were changes that all feared might compromise care, the consensus was that the quality of care remained high and some positive lessons learned.

Multiple changes in oncology services to increase safety were reported, which were largely appreciated by all stakeholders. HPs (but not patients and family members) were concerned patients might avoid hospital with potential harmful consequences [13]. Indeed, it has been reported that COVID-19 anxiety was the third most common reason for patients postponing chemotherapy [14]. Thus, proactive exploration of patient fears related to COVID-19 and information about the relative risks of delaying consultations and treatment may be required.

Patients, carers and HPs all noted increased stress, with reduced support available, as has been documented elsewhere [6, 15]. Some added strains on participants were perhaps unavoidable during a pandemic but highlight the need for preparation and resourcing. Creative ways to provide support to patients and carers through social media and online sources are required [16]. Hlubocky et al. [6] have advocated for healthcare organisations to better support oncology staff by physically protecting them, providing truthful, transparent COVID-19 care information, preventing situations that could cause moral distress and providing staff assistance programmes.

All three stakeholder groups noted challenges with telehealth, although finding it also had benefits (such as reducing travel). Additional administrative layers and communication barriers involved in telehealth should be addressed to avoid increasing disparity; patients from lower socio-economic backgrounds and with poorer health or technology literacy struggle more with this healthcare approach. Burbery et al. [17] provide useful guidelines to optimise the delivery of telehealth, including careful selection of a secure communication platform that facilitates good communication and sharing of information and ensuring adequate lighting and a noise-free environment. They recommmend targeted questioning to encourage disclosure of emotions and symptoms. Non-exclusive use of telehealth as an adjunct to face-to-face communication, with consideration of patient preference, clinical factors and practical issues such as patient ability to travel, is encouraged. The American Society of Clinical Oncology has also published relevant guidelines [18], with comprehensive advice about when and how often telehealth is appropriate, how to put technical assistance in place and how to tailor communication systems to individual patient needs. They note the importance of prioritising the doctor-patient relationship in any exchange. Nevertheless, a comprehensive review of studies evaluating telehealth noted significant gaps in the evidence to guide practice, including studies of patient experience and delivery of active treatment [19]. Furthermore, these authors note the need to train clinicians and patients in use of video prior to its introduction.

All three stakeholder groups emphasised the importance of clear, consistent messaging from trusted sources, individualised to the cancer context. Cancer Australia has responded to this need with excellent resources for oncology health professionals and patients regarding COVID-19 [20]. However, many questions patients, carers and HPs have do not yet have evidence-based answers. Studies are still underway, such as the SerOzNET study, which seeks to better understand the safety and efficacy of COVID-19 vaccines in people with cancer [21]. Future research is needed to fully explore interactions between specific cancer conditions and COVID-19.

While most participants from all stakeholder groups felt the quality of cancer care had not reduced during the COVID-19 pandemic, some HPs noted treatment changes and feared worse outcomes as a result. Supportive care was also cited by all groups as having been impacted, while HPs also noted emergency procedures were impacted. An audit of cancer-related diagnostic and treatment procedures subsidised by the Australian government (MBS items) for five common cancers indeed found initial reductions in total monthly services between March and May 2020, with some recovery over the next few months [22]. A recent systematic review of 62 papers reporting changes in cancer care due to COVID-19 [23] found disruptions to the routine activity of cancer services, reduced cancer surgeries, delayed radiotherapy; and delayed, rescheduled, or cancelled outpatient visits. Reduced personnel (up to 50%) were reported by up to 60% of respondents due to redeployment and quarantine, and a 65% reduction in clinical trials was also reported. Conversely, some HPs in the current study noted that value-based decisions involving cessation of treatment were somewhat easier to make when COVID-19 was factored into the equation and may have resulted in reduced unnecessary treatments. Many colleges and peak bodies internationally are developing guidance for the management of patients with cancer during the COVID-19 pandemic to minimise harm and maximise benefit [24], and these will need careful review as the pandemic develops and vaccination rates increase.

Limitations of the current study include higher rates of university education than in the general population [24], which means results may not represent those held by the wider community of HPs and patient/carers. This is a qualitative study, and further quantitative work is required to examine how widespread are the concerns and experiences discussed by participants.

In conclusion, the primary concerns of all stakeholders engaged in receiving or providing cancer care pertained to safety, increased stress and burnout, communication challenges and the quality of cancer care. As we learn more about COVID-19, ongoing review of guidelines and practices within cancer services will be required to optimise outcomes for all.

## Supplementary information

ESM 1 (DOCX 23 kb)

## Data Availability

The datasets used and/or analysed during the current study are available from the corresponding author upon reasonable request.
