# Supplementary material for: Stakeholder perspectives on the impact of COVID-19 on oncology services: a qualitative study
Source: Support Care Cancer. 2023 Jul 24;31(8):491. doi: 10.1007/s00520-023-07916-y (PMC10366245; doi:10.1007/s00520-023-07916-y)
Supplement: Supplementary file 1 — (DOCX 23 kb) [file 520_2023_7916_MOESM1_ESM.docx]

Supplementary Table 1. Additional quotes.

1. **Safety**

*"The amount of procedures you have to go through to get in: the checks ins, temperature checks, questions… makes me feel fairly secure… the hospital's doing everything it can to keep everyone safe. " (P328)*

*“you’re dealing with someone who’s, an oncologist who is very aware of infection control anyway.” (402)*

*“We didn't have temperatures taken or anything… I thought a little bit casual.” (P340)*

*“The ones just going for their three-month or six-month check were too frightened to go [to hospital].” (NGO212)*

1. **Increased stress and burnout**

*“When I was in hospital during my chemo, there was one… 12-day stay when nobody could visit me at all.” (P333)*

*“Their [carers’]anxiety levels are often much higher than the person who has cancer… because they don't have the care team …, interacting with them all the time, so they're somewhat left out of it.” (HP127)*

*“…a lot of chaos and disruption generally… time being taken up with lots of urgent attention. What we were going to do, how we were going to manage patients, more about thinking and planning into the future rather than what was happening in the here and now.” (HP101)*

*“The information side made the day a bit harder, not because you didn't want the information or didn't want to impart the information… an extra burden in terms of getting that and achieving your workload as well.” (HP508)*

*“So… do you try and organize scans, for example, closer to home and then have a telehealth to get the results, but…you've got to make sure that the scans and results are available in time for that consult. So, there's another admin layer added.” (HP126)*

*“I found I couldn't do any more than three back-to-back [telehealth] sessions because I was just exhausted.” (105)*

*“…there’s been a couple of times where there's been more people calling in sick… doctors never call in sick!… Or if there's been an outbreak in a certain ward… the oncology team might have to take on extra patients who aren't oncology patients...” (HP148)*

*“I was just constantly flitting between nursing home appointments, work schedules, weekend rounds, ensuring I was doing all that was requested of me from a policy and administrative level as well as medical students, and junior staff doing their training as well as patient care, particular… trying to keep outpatients safe as well, and patients managed and micromanaging the ward.” (HP508)*

*“…we compliment so much of what's going on within the health care system… my peer support programmes… the practical support and the financial assistance. For social workers, psychologists, care coordinators in cancer treatment centres, it's another lifeline to some of their patients that they just default (to). So what will happen if we go out of business?” (206)*

*“So, we were asked if we would be willing to have a 10 percent reduction in our base pay, which I personally agreed to. Then all of the programmes were reviewed in April and some of the programmes were cut back to either a day or two a week..” (208)*

1. **Communication challenges and gains**

**3.1 Information**

*“Look, I don’t recall anything particularly to be honest, apart from…. having your temperature taken and questions asked and forms to fill in… I don’t actually recall having any discussion about COVID-19 with any of the oncologist people themselves. (P343)*

*“I don’t feel like there was any information about how to manage cancer and COVID-19 simultaneously.” (P328)*

*“At one point there were emails coming from multiple sources every day, and it's just over the top and especially if there's conflicting messages.” (HP106)*

*And the ethical and medico-legal aspects of it… Should I dose reduce chemotherapy because they're at greater risk?” (HP106)*

**3.2 Communication quality**

*“But the level of professionalism, the caring, the compassion, the kindness from all the professionals I've interacted with, the radiologist, the MRI people and everyone, it's blown me away. “(P333)*

*"With cancer treatment, I'm pretty forgetful… and that's hard enough when you're talking to somebody in person. But when you're on the phone, those things slip in and out much more quickly, I think. " (P328)*

*“…I've actually quite liked it [telehealth] because we're able to offer a bit more support to regional patients, patients who can't get into the hospital for other reasons.” (HP105)*

1. **Quality of care.**

*“He used to have immunotherapy before the operation every two weeks. And after the operation, the oncologist… said a lot of people are changing it to monthly because of Covid-19... But he said, either way, there's no real harm.” (C407)*

*“…we developed a sort of a position statement… for example… the following group of patients should move from intravenous to oral chemotherapy… the following group should probably have a break from chemotherapy. And we shouldn't be doing bone marrow transplants for this group.” (HP102)*

*“We have some ability to do chemotherapy at home depending on the patient, depending on the hospital, depending on the oncologist… that has worked for some people.” (HP113)*

*“And I think before COVID – sometimes we do give chemotherapy that maybe we shouldn't give, and I've been doing much less of that. And that's probably a good thing... COVID is a nice cover to say no you can’t have it” (HP148)*

*“In … patients with advanced stage cancer, depending on what else is going on, it would be more challenging sometimes for them to access treatment or even experimental treatments like clinical trials.” (HP122)*

*“He would have the blood test and then the doctor would ring and just talk to him about how he felt and how his blood tests went and then say, yes, you're okay to start again tomorrow…. So, we weren't touching base at the hospital at all. And after about four months of that, I began to feel a bit nervous. I thought, he's not a person who's very in touch with his body.” (C406)*

*“Since it's been Telehealth, even though it's Zoom, they don't do any of that assessment. They just ask him how he's been and review the medication. He'll say, we'll see you in another six months. So it's much reduced that service, much more efficient, but much reduced.” (C413)*

*“If I'm in the consultation and I hear exactly what he's hearing. I can know whether or not he's downplaying something or whether it's accurate.” (C413)*
